# Differential Expression of miR-223-3p and miR-26-5p According to Different Stages of Mastitis in Dairy Cows

**DOI:** 10.3390/biom15020235

**Published:** 2025-02-06

**Authors:** Eleonora Dall’Olio, Fabio De Rensis, Eugenio Martignani, Silvia Miretti, Ugo Ala, Valeria Cavalli, Claudio Cipolat-Gotet, Melania Andrani, Mario Baratta, Roberta Saleri

**Affiliations:** 1 Department of Veterinary Science, University of Parma, Strada del Taglio 10, 43126 Parma, Italy; eleonora.dallolio@unipr.it (E.D.); fabio.derensis@unipr.it (F.D.R.); valeria.cavalli@unipr.it (V.C.); claudio.cipolatgotet@unipr.it (C.C.-G.); melania.andrani@unipr.it (M.A.); roberta.saleri@unipr.it (R.S.); 2 Department of Veterinary Sciences, University of Torino, Largo Paolo Braccini 2, 10095 Grugliasco, Italy; eugenio.martignani@unito.it (E.M.); silvia.miretti@unito.it (S.M.); ugo.ala@unito.it (U.A.); 3 Department of Chemistry, Life Sciences and Environmental Sustainability, University of Parma, Parco Area delle Scienze 11a, 43124 Parma, Italy

**Keywords:** mastitis, dairy cows, biomarkers, differential somatic cell count, miR-223, miR-26

## Abstract

Mastitis is the leading cause of economic losses in dairy farming, significantly impairing animal welfare and the quality and quantity of milk production. MicroRNAs are increasingly gaining attention, in both human and veterinary medicine, as biomarkers for various diseases. This study evaluated the diagnostic potential of four circulating microRNAs (miR-26-5p, miR-142-5p, miR-146a, and miR-223-3p) by examining changes in their expression in milk samples from dairy cows at different immune-cell subpopulations correlated to different stage of mastitis with a validated method. Additionally, this study has analyzed the possible source of these circulating microRNAs by the measurement of their secretion from activated immune cells (lymphocytes, monocytes, and neutrophils). miR-223-3p has been significantly expressed in an acute stage of mastitis (*p* < 0.01) but not in the chronic or susceptible stages. Conversely, mir-26-5p has been significantly reduced in acute, chronic, and susceptible groups of animals. In immune-cell cultures, miR-26 has been shown to be down-regulated in lipopolysaccharide (LPS)-stimulated neutrophils, while miR-223 has been shown to be up-regulated in phytohemagglutinin (PHA)-stimulated lymphocytes. The differential expression of miR-223-3p and miR-26-5p, combined with differential and total somatic cell count, could serve as a useful tool for identifying the evolutionary stage of mastitis-related inflammatory pathology.

## 1. Introduction

Mastitis is a disease that significantly affects cow welfare and causes substantial economic losses to dairy herds, both in terms of milk yield losses and treatment costs [1,2]. Indeed, mastitis leads to a reduction in milk production and quality, and it is one of the primary reasons for the use of antimicrobial treatments in the dairy industry [2,3]. Given concerns regarding antibiotic resistance and the need to minimize treatment costs, the early diagnosis of mastitis in dairy cows, both in clinical and subclinical forms, is becoming increasingly relevant.

Mastitis is classified as either clinical or subclinical, with diagnosis primarily based on direct or indirect somatic cell count (SCC), despite its lower accuracy than microbiological analysis. Currently, the recognized SCC threshold for a clinical mastitis diagnosis is 4 × 10^5^ cells/mL of milk, while the reference threshold for subclinical mastitis is 2 × 10^5^ cells/mL [4,5]. Although SCC has always been an excellent indicator of the bovine udder health status, its reliability is decreasing; indeed, it has been observed that mammary gland inflammation can even have an SCC lower than 1 × 10^5^ cells/mL [6]. An alternative widely used in the field is the California Mastitis Test (CMT), which, however, has inherent limitations due to the subjectivity of the operator performing the test.

Somatic cells in milk consist of epithelial and immune cells, with their proportions varying based on different factors, such as physiological factors (e.g., lactation stage) and pathological conditions (e.g., inflammation). Recently, differential somatic cell count (DSCC) has emerged as a more refined indicator of udder inflammation. Unlike SCC, DSCC provides a qualitative assessment by determining the percentage of immune cells, specifically lymphocytes and neutrophils. A DSCC threshold of 68.5% is used to differentiate healthy cows from those affected by mastitis [3,6,7,8]. Lymphocytes regulate the initiation and suppression of the immune response in case of infection, while circulating monocytes play a crucial role in innate immunity and become resident macrophages upon migration into tissues. Type 1 macrophages (M1) perform a pro-inflammatory action by releasing inflammatory mediators (such as IL-1, IL-6, and TNF-α), whereas type 2 macrophages (M2) possess phagocytic activity to eliminate pathogens and cellular debris [9,10,11]. Additionally, macrophages recognize pathogens and rapidly recruit neutrophils to the site of infection [10]. Neutrophils also have high phagocytic activity and release enzymes capable of killing pathogens [12].

In healthy cows, milk somatic cells are primarily represented by lymphocytes (75%), followed by monocytes/macrophages (18%), neutrophils (5%), and epithelial cells (2%). However, this composition changes significantly in cases of clinical or subclinical mastitis. In clinical mastitis, the proportion of neutrophils increases drastically (55%), while monocytes/macrophages rise to 36% and lymphocytes decrease to 6%. A similar trend is observed in subclinical mastitis, with neutrophils increasing to 65%, lymphocytes dropping to 3%, and monocytes/macrophages reaching 28% [3,10,13]. DSCC, when combined with SCC, enhances diagnostic accuracy. However, detecting the described changes in the composition of somatic cells in milk shows a certain delay compared to the onset of infection [3]. Therefore, it is necessary to identify new methods that allow for the early diagnosis of mastitis, both in its clinical and subclinical forms.

MicroRNA (miRNA) are non-coding RNA molecules approximately 20–22 nucleotides long that regulate gene expression at the post-transcriptional level [14]. miRNAs can be released from cells in response to both physiological and pathological stimuli into body fluids, including milk, and in this context, they are referred to as circulating miRNAs (c-miRNAs). Interestingly, these c-miRNAs in milk show high resistance to multiple freeze–thaw cycles and the action of RNases [15]. This high stability makes them excellent molecules to work with. Additionally, several miRNAs have been associated with inflammatory processes, and some have shown alterations in their expression related to mastitis in dairy cows, making them interesting candidates as diagnostic biomarkers [16,17,18,19].

This study aims to evaluate the alterations in the expression of a set of four c-miRNAs, previously associated with bovine mastitis (miR-26a-5p, miR-142-5p, miR-146a, and miR-223-3p) [20], in milk samples in relation to the inflammatory state of the mammary gland of dairy cows. The goal is to assess their potential as biomarkers for the early diagnosis of mastitis and to investigate their secretion dynamics in relation to specific immune cell populations in milk.

## 2. Materials and Methods

### 2.1. Ethical Statement

The project has been approved by the Ethics Commission for Animal Experimentation (ECAE) of the University of Parma (prot.02/CESA/2025). All the dairy cows involved in this study were reared in commercial private farms and were not subjected to any invasive procedures. Milk samples used for the analyses were collected during routine milking. All milk samples were collected and analyzed according to the guidelines for dairy cattle milk recording and analysis [21].

### 2.2. Milk Samples

Milk samples used in this study were collected from 72 Italian Brown Swiss dairy cows (5.1 ± 1.6 years old) screened for SCC and DSCC composition. Briefly, milk samples were divided depending on neutrophil and lymphocyte composition and the clinical status of the mammary gland according to the status of mammary inflammation. Two liters of milk per cow were sampled from the evening milking sessions between February 2021 and April 2022 and stored at 4 °C immediately after collection. The samples were analyzed within 24 h and subsequently aliquoted into 2 mL Eppendorf tubes and stored at −20 °C for subsequent microRNA analyses. Each sample was analyzed for fat, protein, lactose, and casein content using a MilkoScan FT3 (Foss Electric A/S, Hillerød, Denmark), while SCC and DSCC (neutrophils + lymphocytes, %) were determined using a Fossomatic 7DC (Foss Electric A/S, Hillerød, Denmark). The 72 milk samples were divided into four groups of 18 samples, each referring to a validated procedure based on the interaction between SCC and DSCC, as described by Stocco et al. [7,8]. Based on the thresholds of 2 × 10^5^ somatic cells/mL for SCC and 68.5% for DSCC as reference points, the obtained data allowed for subdivision into four experimental groups. Cows with mean SCC and DSCC values below both thresholds were classified as the control group (CTRL), while those with SCC and DSCC values above both thresholds were classified as the acute mastitis group (AM). Cows with SCC > 2 × 10^5^ somatic cells/mL and DSCC < 68.5% were classified as the chronic mastitis group (CM), while those with SCC < 2 × 10^5^ somatic cells/mL and DSCC > 68.5% were identified as the susceptible group (SU) (Table 1).

### 2.3. Circulating microRNA Extraction and Reverse Transcription

A total of 72 animals were included in this study according to the four differential immune-cell compositions in milk previously described. C-miRNAs were extracted from milk samples using the Maxwell^®^ RSC miRNA from Plasma and Serum Kit (Promega, Madison, WI, USA), an automated system for obtaining high-quality total RNA with enhanced miRNA enrichment on a Maxwell^®^ RSC Instrument (Promega, Madison, WI, USA).

c-miRNAs from the immune-cell culture mediums were extracted using a protocol with TRIzol^®^ Reagent (Life Technologies, Waltham, MA, USA) as described by Ioannidis et al. [22]. RNA samples were immediately reverse transcribed to complementary DNA (cDNA) using the miRCURY^®^ LNA^®^ RT Kit (Qiagen, Milan, Italy). Extractions yield 4 ng of total RNA. RT was performed using a StepOne™ thermocycler (Applied Biosystems, StepOne™ software v.2.3; Waltham, MA, USA) according to the manufacturer instructions under the following thermal conditions: 1 h at 42 °C, followed by 5 min at 94 °C. For the quality control of extraction and reverse transcription, synthetic RNA spike-ins UniSp-2,4,5 template mix from the RNA Spike-In Kit, For RT (Qiagen, Milan, Italy) and UniSp-6 from the miRCURY^®^ LNA^®^ RT Kit (Qiagen, Milan, Italy) were used, respectively. The cDNA samples were stored at −20 °C until used.

### 2.4. Gene-Expression Analysis

For the analysis of c-miRNA expression, cDNA templates were subjected to real-time quantitative PCR (qPCR), carried out in a StepOne™ thermocycler (Applied Biosystems, StepOne™ software v.2.3; Waltham, MA, USA). The diluted cDNA (1:30) was amplified in a volume of 10 μL in duplicates using the miRCURY LNA SYBR^®^ Green PCR Kit (Qiagen, Milan, Italy) and specific primers for bovine sequences of miR-26a-5p (efficiency: 98.4%), miR-142-5p (efficiency: 98.6%), miR-146a (efficiency: 101.2%), miR-148a-3p (efficiency: 98.8%), or miR-223-3p (efficiency: 98.9%; Qiagen, Milan, Italy), with UniSp-2,4,5 and -6 for synthetic sequences (Qiagen, Milan, Italy). Samples were kept at 95 °C for 2 min and then subjected to 40 cycles consisting of a denaturation step at 95 °C for 10 s, followed by an annealing step at 56 °C for 60 s. A melting-curve analysis was performed (60–95 °C) at the end of the amplification cycles. Data were analyzed using the 2^−ΔΔCq^ method, in which the expression levels of each gene were expressed as fold change normalized to the reference gene miR-148a-3p, a highly abundant c-miRNA constitutively expressed in dairy milk [20,23,24] as the endogenous control and to the exogenous control, according to Tzelos et al. [20]. A cut-off value in relative gene expression (fold change) was set at 1.5 and 0.67 for up-regulation and down-regulation, respectively, as other authors have reported for exploratory studies [25].

### 2.5. Isolation of Bovine Lymphocytes, Monocytes, and Neutrophils

Peripheral blood mononuclear cells (PBMCs) and neutrophils were isolated from bovine blood provided by a slaughterhouse certified by the Italian Ministry of Health in accordance with European Regulation (EC) 853/2004 approval nr. CE-IT-218-M). The isolation was performed using a density gradient protocol with Histopaque-1.077^®^ (Merck; Darmstadt, Germany) as described in Ferrari et al. [26] for PBMCs and modifying the protocol described by Kouoh et al. [27] for neutrophils. After isolation, PBMCs were incubated at 37 °C, 5% CO_2_ for 24 h to allow for the complete adhesion of monocytes to the flask in cRPMI-1640 + 10% of fetal bovine serum (FBS). The monocytes were washed twice with PBS to remove all non-adherent cells that might have remained in the flask. After removing PBS, the monocytes were incubated with 0.25% trypsin-EDTA (Gibco, Grand Island, NY, USA) for 5–10 min at 37 °C, 5% CO_2_. After incubation, cRPMI-1640 + 10% FBS was added to stop the action of trypsin, and the monocytes were centrifuged and washed twice with the same culture medium.

For neutrophil isolation, 5 mL of EDTA-anticoagulated blood was layered onto Histopaque-1.077^®^, and after centrifugation, only the erythrocyte layer containing neutrophils was retained. Following erythrocyte lysis, neutrophils with a purity >95% were obtained. Cells purity was assessed after Diff Quick staining by performing a differential blood cell count of isolated cells’ smear. Neutrophils were incubated at 37 °C, 5% CO_2_ for 1 h in cRPMI-1640 + 10% FBS to allow them to adjust to new conditions, given their extreme sensitivity even to minor mechanical stimuli [28].

After being washed twice with cRPMI-1640 + 10% FBS (lymphocytes and monocytes) or with Hanks Balanced Salt Solution (HBSS) without calcium, magnesium, and phenol red (neutrophils), immune cells were seeded at a density of 2 × 10^5^ cells/well in 24-well plates, and viability was checked with Trypan Blue (never less than 95%).

### 2.6. Inflammatory Stimulation of Bovine Immune-Cell Cultures

Lymphocytes were stimulated for 4 h or 24 h with 5 μg/mL of phytohemagglutinin (PHA, from Phaseolus vulgaris; Sigma-Aldrich, Darmstadt, Germany) or were maintained in a control condition in cRPMI-1640 + 10% FBS. Neutrophils and monocytes were stimulated for 4 h or 24 h with 1 μg/mL of lipopolysaccharide (LPS, from pathogenic *E. coli* serotype 0111:B4; Merck, Darmstadt, Germany) or were maintained in a control condition in cRPMI-1640 + 10% FBS.

After the incubation with PHA or LPS, the culture medium from immune-cell cultures was collected and frozen at −20 °C from each well of the 24-well plates for subsequent microRNA analyses.

### 2.7. Statistical Analysis

The sample size calculation for 4 groups of milk samples was carried out through the “pwr” package (version 1.3-0) as implemented in R (version 4.3.3) and Rstudio (release 2023.06.1) environment. The prediction was based on requiring a significance level of 0.05, a statistical power of 80%, and relying on a prediction of a large effect size, set with a value of 0.4.

c-miRNAs’ expression profiles have been compared among groups through a Kruskal–Wallis rank sum test and subsequent pairwise comparisons using a Wilcoxon rank sum test with continuity correction and *p* value adjustment by the Benjamani–Hochberg method. Correlations between miRNAs and between qPCR technique normalization methods have been based on Spearman’s rank correlation coefficient rho.

## 3. Results

### 3.1. SCC and DSCC in Milk Samples

**Figure 1:**
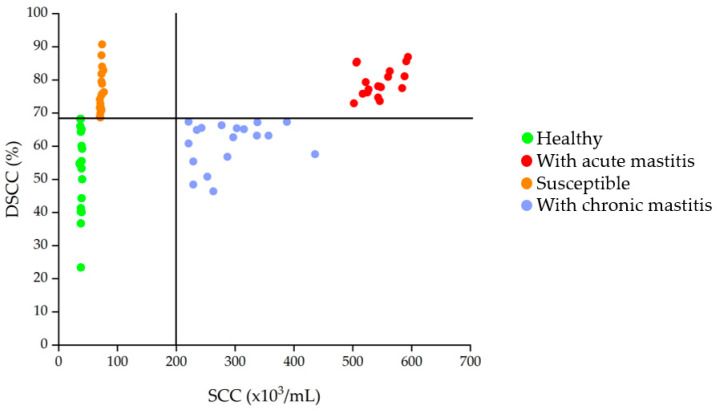
Scatter plot showing the distribution of dairy cows based on the two thresholds for somatic cell count (2 × 10^5^ somatic cells/mL) and differential somatic cell count (68.5%).

Following analysis with the Fossomatic 7DC (Foss Electric A/S, Hillerød, Denmark), data on SCC and DSCC were obtained from milk samples. These data enabled the definition of the four experimental groups for the in vivo study, as previously reported. The mean values of collected data for each experimental cow regarding parity, daily milk yield, and days in milk (DIM) are shown in Table 2. There were no differences in these factors across groups. Additionally, the scatter plot illustrating the distribution across groups of dairy cows is shown in Figure 1. The mean values obtained from milk composition analysis and the mean SCC and DSCC values divided by group are reported in Table 3.

### 3.2. c-miRNA Expression in Milk

**Figure 2:**
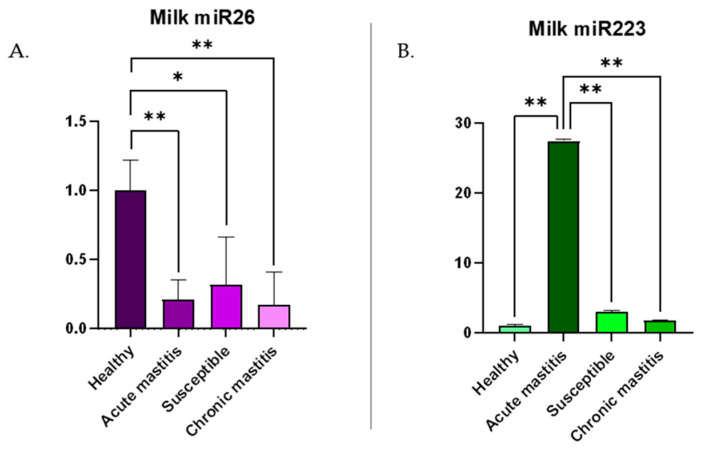
Relative expression levels reported as mean ± SD of (**A**) bta-miR-26a-5p and (**B**) bta-miR-223-3p in milk samples from each group. Data were analyzed using the 2^−ΔΔCq^ method, in which the expression levels of the gene have been normalized to the expression of the reference genes bta-miR-148a-3p and UniSp2. * indicates a significant difference with a *p*-value < 0.05; ** indicates a significant difference with a *p*-value < 0.01. CTRL = control, AM = with acute mastitis, SU = susceptible, CM = with chronic mastitis.

miR-26a-5p (miR-26) showed down-regulation in samples from cows with acute mastitis (0.8 fold, *p* < 0.01), susceptible (0.7 fold, *p* < 0.05), and with chronic mastitis (0.8 fold, *p* < 0.01) compared to the control group, as shown in Figure 2A. miR-223-3p (miR-223) was up-regulated in samples from animals with acute mastitis compared to control (26.5 fold, *p* < 0.01), susceptible cows (24.4 fold, *p* < 0.01), or those with chronic mastitis (25.7 fold, *p* < 0.01), as shown in Figure 2B. As reported in Table 1, the groups were identified based on the threshold values of SCC (2 × 10^5^ cells/mL) and DSCC (68.5%) according to the following criteria: the CTRL group showed SCC and DSCC values below their respective thresholds; the SU group had SCC values below the threshold but DSCC above 68.5%; the AM group had values exceeding both thresholds; the CM group exceeded the SCC threshold but not the DSCC threshold. The mean ± SD values for SCC and DSCC per group are available in Table 3.

**Figure 3:**
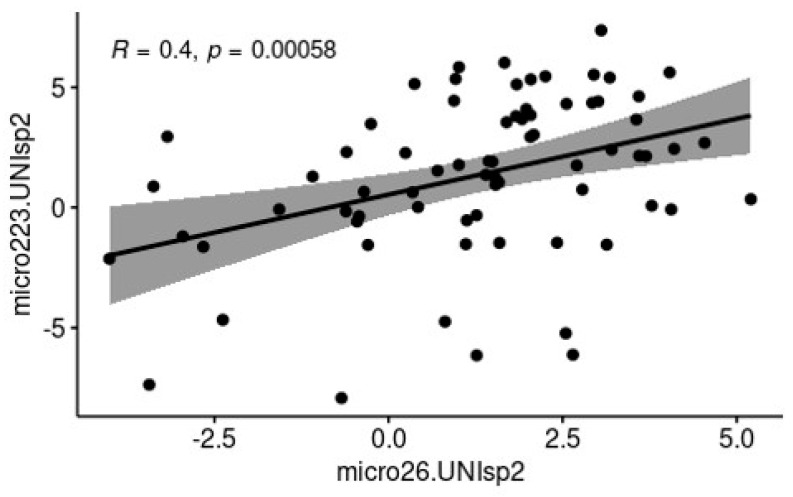
Correlation between bta-miR-223 and bta-miR-26. Data have been normalized to the expression of the reference genes bta-miR-148a-3p and UniSp2. R and *p*-value are displayed on the graphs.

A positive correlation between the two c-miRNAs, miR-223 and miR-26, was also observed for data normalized to both the endogenous and exogenous controls (r = 0.4, *p* < 0.01), as shown in Figure 3.

miR-142-5p (miR-142) and miR-146a (miR-146) were excluded from the study after an initial analysis of milk samples, as they did not show any differences between groups.

### 3.3. c-miRNA Expression in Immune Cells

**Figure 4:**
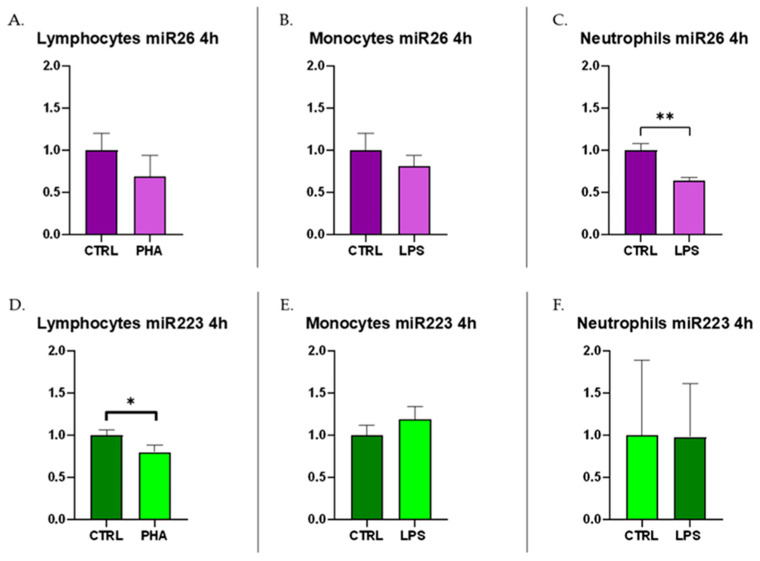
Relative expression levels reported as mean ± SD of bta-miR-26a-5p in (**A**) lymphocytes, (**B**) monocytes, (**C**) neutrophils, and of bta-miR-223-3p in (**D**) lymphocytes, (**E**) monocytes, and (**F**) neutrophils’ culture medium, unstimulated or stimulated for 4 h with PHA or LPS. Each group includes samples of 3 replicates from three separate experiments. Data were analyzed using the 2^−ΔΔCq^ method, in which the expression levels of the gene have been normalized to the expression of the reference genes bta-miR-148a-3p and UniSp2. * indicates a significant difference with a *p*-value < 0.05; ** indicates a significant difference with a *p*-value < 0.01.

Regarding miR-26, lymphocytes and monocytes showed no differences between the control and PHA- or LPS-stimulated groups, respectively (Figure 4A,B). However, the down-regulation of miR-26 was observed in neutrophils after the 4 h LPS stimulation compared to the control group (*p* < 0.01), as shown in Figure 4C.

miR-223 showed a down-regulation in lymphocytes stimulated with PHA for 4 h (*p* < 0.05) compared to the control group (Figure 4D). In monocytes and neutrophils, no differences were observed between the control group and the group stimulated with LPS for 4 h (Figure 4E,F). A tendency towards up-regulation can only be observed in the monocytes’ culture after 4 h of LPS stimulation.

## 4. Discussion

The primary aim of this study was to evaluate the expression alterations of four c-miRNAs (miR-26-5p, miR-142-5p, miR-146a, and miR-223-3p) in milk samples in relation to different stages of the inflammatory process caused by mastitis in dairy cows. Subsequently, their expression was assessed in the culture medium of activated immune cells. Recently, it has been reported that milk fat miRNome changes in response to LPS challenge in Holstein cows [29]. A previous study demonstrated that miR-26 expressed in mammary gland tissue of donkeys and goats appears to target mRNAs involved in immunity, as well as genes associated with fatty acid biosynthesis and members of the PI3K-Akt and MAPK pathways, resulting, among other effects, in increased resistance to apoptosis [30]. An increase in miR-26 expression can inhibit the hyperglycemia-induced overexpression of PFKFB3 [31]. Moreover, miR-26 has been observed to induce increased expression of cytokines and chemokines, highlighting its involvement in the inflammatory process regulation [32]. To date, no studies have yet clarified the role of miR-26 in bovine mammary gland tissue and milk. Recent studies have observed a modest increase in miR-26 levels in the blood of dairy cows during early pregnancy [33]. Additionally, its expression in bovine milk has been correlated with the CMT score and lactation stage, showing higher expression during the transition from CMT score 0 to 1 and in cows during their first lactation [20]. Finally, it has been reported that the molecules secreted by *S. aureus* can modulate the immune response of bovine leukocytes in vitro, highlighting how secretomes from *S. aureus* strains with different epidemiological behaviors could elicit dramatically different responses in bovine PBMCs [34]. Our study has collected some preliminary data on the possible origin of the secretion of these microRNAs found in milk. The cells that can be found in milk during mastitis are, in addition to the luminal and myoepithelial cells of the alveolus, the stromal cells and, especially, the immune cells that we have characterized as lymphocytes, neutrophils, and monocytes. If activated, these cells confirm their ability to modulate the microRNAs studied. Therefore, we can hypothesize that such cells might be partially responsible for the changes in miRNA levels that we detected in vivo in clinical or subclinical mastitis. Since variations in milk miRNA content are higher than what we detected in our in vitro model; further studies will be necessary to evaluate the role of other cell types involved, such as other somatic cells or even cells belonging to the mammary parenchyma.

In the present study, miR-26 exhibited a significant down-regulation in milk during inflammation (Figure 2A), showing markedly reduced levels in groups with acute or chronic mastitis, as well as in the group of susceptible cows, which were in the very early stages of an inflammatory process at the time of sampling. Considering that miR-26 is ubiquitously expressed in the body and is among the most abundant c-miRNAs in milk and mammary tissues [20], its expression in the culture medium of immune cells is also noteworthy. miR-26 showed a significant down-regulation in neutrophils activated with LPS as early as 4 h after stimulation, and a trend toward reduction was also observed in the activated lymphocytes and monocytes (Figure 4A–C). The reduced expression of miR-26 in both matrices aligns with previous findings, which have already demonstrated a significant decrease in miR-26 expression during inflammatory processes [31,32].

miR-142 has been more extensively studied in bovine mastitis, and its overexpression has been observed in milk, blood, and mammary gland epithelial cells [20,35,36]. Previous studies have shown that through the activation of the NF-κB signaling pathway, miR-142 induces the release of cytokines such as TNF-α, IL-1β, IL-6, and IL-8, suggesting its pro-inflammatory role in the mastitis process [35,36,37]. miR-146 has also been studied in bovine mastitis, and it has been shown to play a negative feedback role in the inflammatory process by down-regulating the Toll-Like Receptor 4/TNF Receptor-Associated Factor 6/NF-κB (TLR4/TRAF6/NF-κB) pathway [38]. A significant increase in miR-146 has been observed in the mammary gland tissues of dairy cows with mastitis, but no variation was observed in blood samples [39]. In the present study, both miR-142 and miR-146, after initial analysis, did not show any differences between groups in milk samples.

miR-223 regulates the production and activation of granulocytes by exerting a negative control on the proliferation of progenitors as well as their differentiation and activation [40,41]. It also regulates the differentiation and proliferation of dendritic cells and macrophages, regulating their inflammatory or anti-inflammatory polarization. Specifically, the overexpression of this c-miRNA promotes M2 macrophage polarization, while reduced expression leads to M1 macrophage polarization [42]. Moreover, miR-223 has been shown to regulate the inflammatory process by promoting the proliferation of T helper cells, inhibiting the release of inflammatory mediators, and blocking inflammatory signaling pathways, such as the NLRP3 inflammasome and NF-κB signaling pathways [42,43,44].

The role of miR-223 in mastitis remains to be fully elucidated, but recent studies have investigated this c-miRNA in bovine mastitis. miR-223 appears to participate in the TLR signaling pathway, leading to the reduced transcription and secretion of IL-1, IL-6, and IL-8, while activating innate immunity during mastitis pathogenesis, thereby mitigating inflammation [42,45]. Zhou et al. [46] demonstrated that miR-223 down-regulates the NLRP3 inflammasome and the Keap1/Nrf2 signaling pathway, thereby reducing inflammation and oxidative damage associated with the mastitis process. This anti-inflammatory regulatory feature makes miR-223 a potential candidate for the development of novel treatments for bovine mastitis and other inflammatory diseases [46]. In mice with LPS-induced inflammation of mammary epithelial cells, an increase in miR-223 expression has been observed [41]. This up-regulation has also been reported in the mammary tissue of cows with mastitis, as well as in bovine mammary epithelial cells (bMECs), granulocytes, dendritic cells, T cells, endothelial cells, and epithelial cells during inflammation [45,47,48,49]. Consistent with previous findings, the present study observed a significant up-regulation (*p* < 0.01) of miR-223 (Figure 2B) in milk samples from dairy cows with acute mastitis compared to the control group, as well as to the chronic mastitis and susceptible groups. However, in the lymphocytes, monocytes, and neutrophils’ cultures (Figure 4D–F) stimulated under inflammatory conditions for just 4 h, no increase in miR-223 levels was detected. This is also evident from the results in milk samples, which show a significant and marked increase in cows with acute mastitis, whereas in the susceptible group, no up-regulation is observed compared to controls. This highlights that during the very early stages of the inflammatory process, a significant increase in miR-223 release into body fluids, including milk, has not yet occurred. Srikok et al. [17] also reported a significant increase in miR-223 levels in both serum and whole milk from cows with mastitis, noting that the concentration of this c-miRNA was higher in milk than in serum. Additionally, Tzelos et al. [20] compared the levels of c-miRNAs (miR-26, miR-142, miR-146, miR-223) in whole and skimmed milk, demonstrating that concentrations were higher in whole milk. These findings suggest that whole milk is the optimal matrix for evaluating the presence of miR-223, as well as miR-26. miR-223 expression seems to be different in activated lymphocytes when compared to naive ones. However, the change in expression level is below the cut-off value, so the biological implication is uncertain, and no definitive conclusion can be drawn for the source of this molecule.

Considering the marked up-regulation of miR-223 in the milk of cows in the acute phase of inflammation, miR-223 could be hypothesized as a potential biomarker for the evaluation of mastitis stage in dairy cows and, when combined with SCC or DSCC, it could enhance the accuracy of these widely used diagnostic methods. Furthermore, the observed correlation between miR-223 and miR-26 suggests that the differential evaluation of these two c-miRNAs may provide insights into the progression of inflammation associated with mastitis. miR-26 appears to be responsive in the very early stages of inflammation, as seen in the susceptible group, while miR-223 shows a clear response in animals with acute, clinical, and notably subclinical mastitis, in accordance with the findings of Srikok et al. [17].

## 5. Conclusions

miR-223 and miR-26 are proposed as potential new milk biomarkers that could allow for differential evaluation to identify the developmental stage of the udder inflammation caused by mastitis. The gold-standard method for diagnosing this disease is microbiological analysis, which requires excessive time to complete. The two most used alternatives are SCC and CMT, which, as previously mentioned, face issues related to result reliability and operator subjectivity. For these reasons, there is an increasing need for improved diagnostic tools that may enrich the classification of mastitis in dairy cattle, particularly in the detection of early stages of the illness. Additionally, the origin of these miRNAs’ production remains under investigation. The objective of this study was to assess whether specific subsets of immune-cell populations, particularly lymphocytes and neutrophils, are associated with miRNA production. This study highlighted that miR-26 and miR-223 are synthesized by the subsets of immune cells; further research is undoubtedly needed to determine whether these cells are the primary source of miRNA production within the alveolar environment.

## Figures and Tables

**Table 1:** Division into groups of dairy cows into four categories according to the threshold parameters of SCC and DSCC and their description by group. CTRL = control, SU = susceptible, AM = with acute mastitis, CM = with chronic mastitis.

Group	SCC Value (Cells/mL)	DSCC Value (%)	Description
CTRL	<2 × 10^5^	<68.5	Indicative values of absence of inflammation
SU	<2 × 10^5^	>68.5	Indicative values of initial inflammation resulting in increased susceptibility to mastitis
AM	>2 × 10^5^	>68.5	Indicative values of acute inflammatory state
CM	>2 × 10^5^	<68.5	Indicative values of a chronic decease

**Table 2:** Mean values ± SD of parity, days in milk, and daily milk yield of experimental cows divided by group. CTRL = control, SU = susceptible, AM = with acute mastitis, CM = with chronic mastitis.

Group	Parity	Days in Milk (d)	Milk Yield (kg/Sampling)
CTRL	2.83 ± 1.34	184 ± 108	13.75 ± 4.81
SU	2.56 ± 1.34	205 ± 118	12.44 ± 3.79
AM	3.00 ± 2.14	181 ± 117	11.36 ± 4.38
CM	2.72 ± 1.93	217 ± 107	10.53 ± 3.84

**Table 3:** Mean values ± SD of composition and somatic cell traits of milk samples divided by group. CTRL = control, SU = susceptible, AM = with acute mastitis, CM = with chronic mastitis.

Group	Fat (%)	Protein (%)	Casein (%)	Lactose (%)	SCC (Cells/mL)	DSCC (%)
CTRL	4.06	3.70	2.95	4.85	0.38 × 10^5^ ± 0.01	52.52
SU	4.08	3.72	2.96	4.84	0.73 × 10^5^ ± 0.02	76.73
AM	4.08	3.78	2.98	4.65	5.45 × 10^5^ ± 0.30	79.37
CM	4.39	3.98	3.15	4.62	2.91 × 10^5^ ± 0.62	60.80

## Data Availability

All data are available from the corresponding author upon reasonable request.
